# Supplementary material for: Typing tumors using pathways selected by somatic evolution
Source: Nat Commun. 2018 Oct 8;9:4159. doi: 10.1038/s41467-018-06464-y (PMC6175900; doi:10.1038/s41467-018-06464-y)
Supplement: Supplementary file 1 — Supplementary Information [file 41467_2018_6464_MOESM1_ESM.pdf]

Typing tumors using pathways selected by somatic evolution

Wang et al.

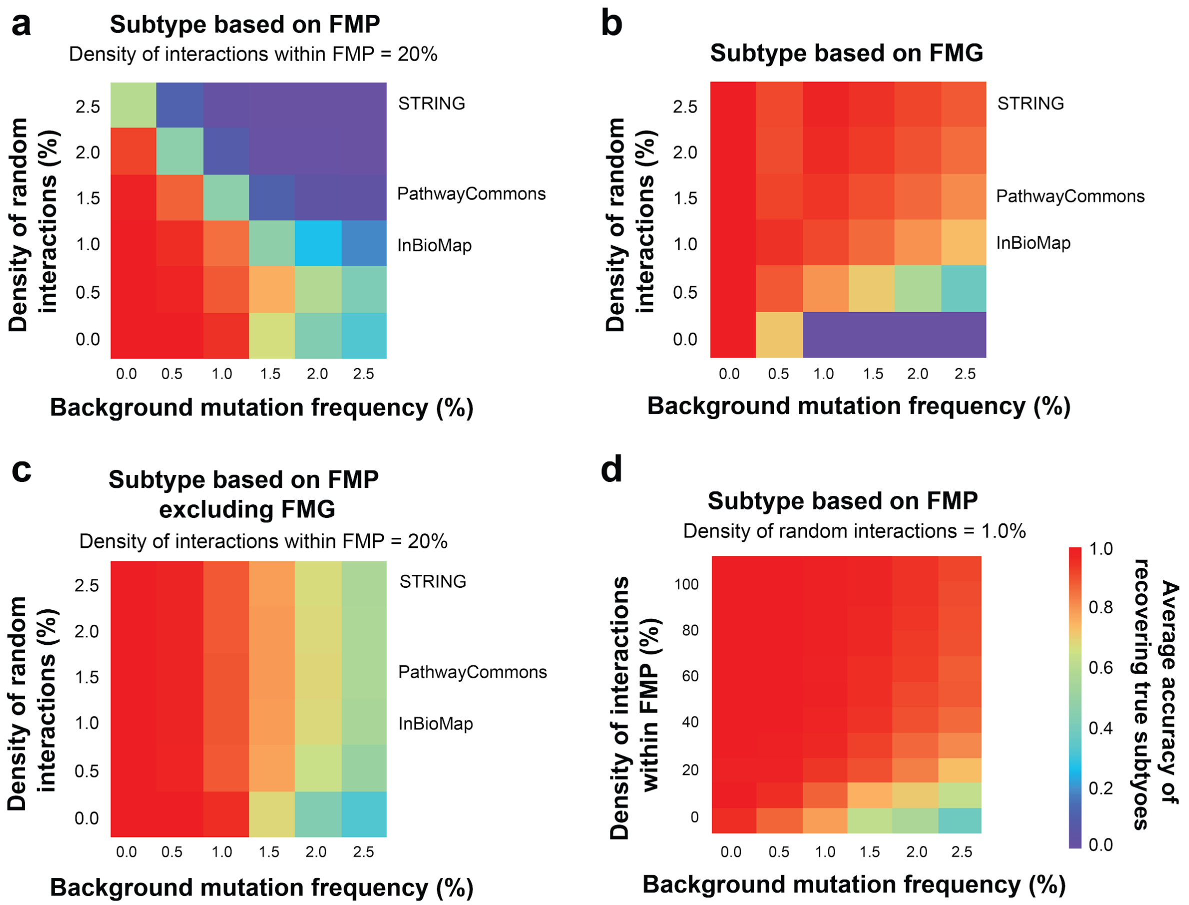

Supplementary Figure 1. Accuracy landscape for different simulations (general densely connected FMP and random edges generated by Erdos-Renyi model). a, ARI landscape across varying background mutation frequency and density of random gene interactions when subtypes are driven by mutations of FMP. **b**, ARI landscape across varying background mutation frequency and density of random gene interactions when subtypes are driven by a FMG. **c**, ARI landscape across varying background mutation frequency and density of random gene interactions when subtypes are driven by mutations of FMP without any FMG presents. **d**, ARI landscape across varying background mutation frequency and density of gene interactions within FMP when subtypes are driven by mutations of FMP.

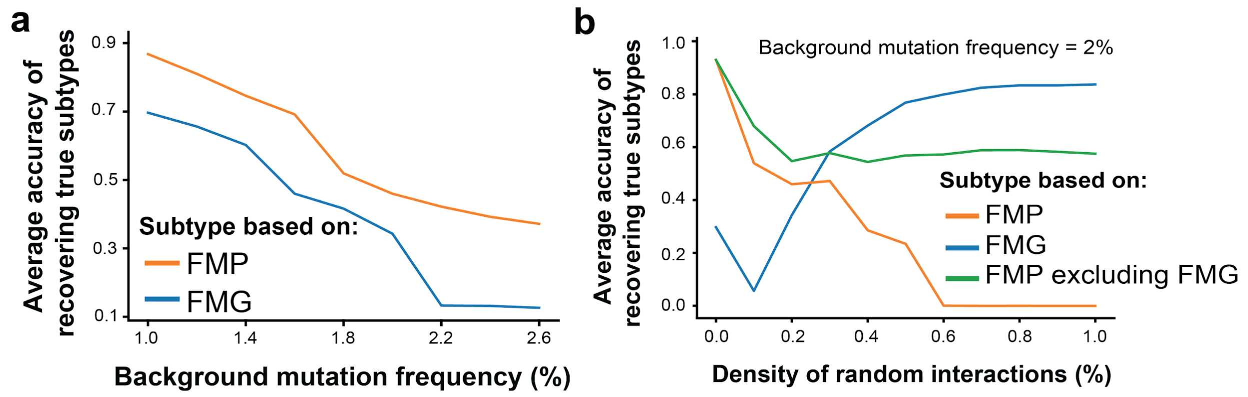

Supplementary Figure 2. Exploring cancer pathway analysis through simulation (star-like FMP and random edges generated by preferential attachment model). a, Tumor stratification performance with increasing frequency of background mutations, when no random interactions are presented. Performance is measured by calculating Adjusted Random Index (ARI) between the true subtypes and the tumor clusters derived by network stratification. **b,** Tumor stratification performance with increasing random gene interaction density.

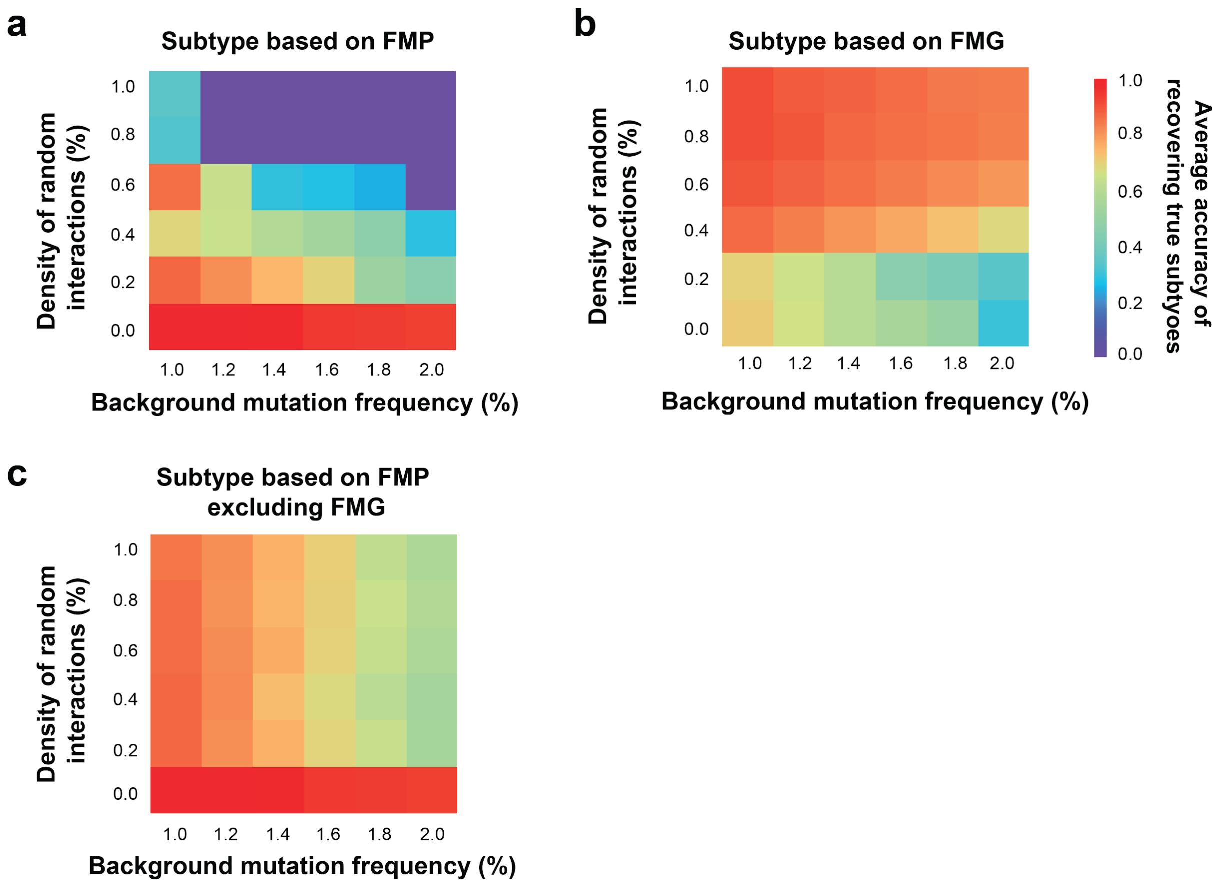

Supplementary Figure 3. Accuracy landscape for different simulations (star-like FMP and random edges generated by preferential attachment model). a, ARI landscape across

varying background mutation frequency and density of random gene interactions when subtypes are driven by mutations of FMP. **b**, ARI landscape across varying background mutation frequency and density of random gene interactions when subtypes are driven by a FMG. **c**, ARI landscape across varying background mutation frequency and density of random gene interactions when subtypes are driven by mutations of FMP without any FMG presents.

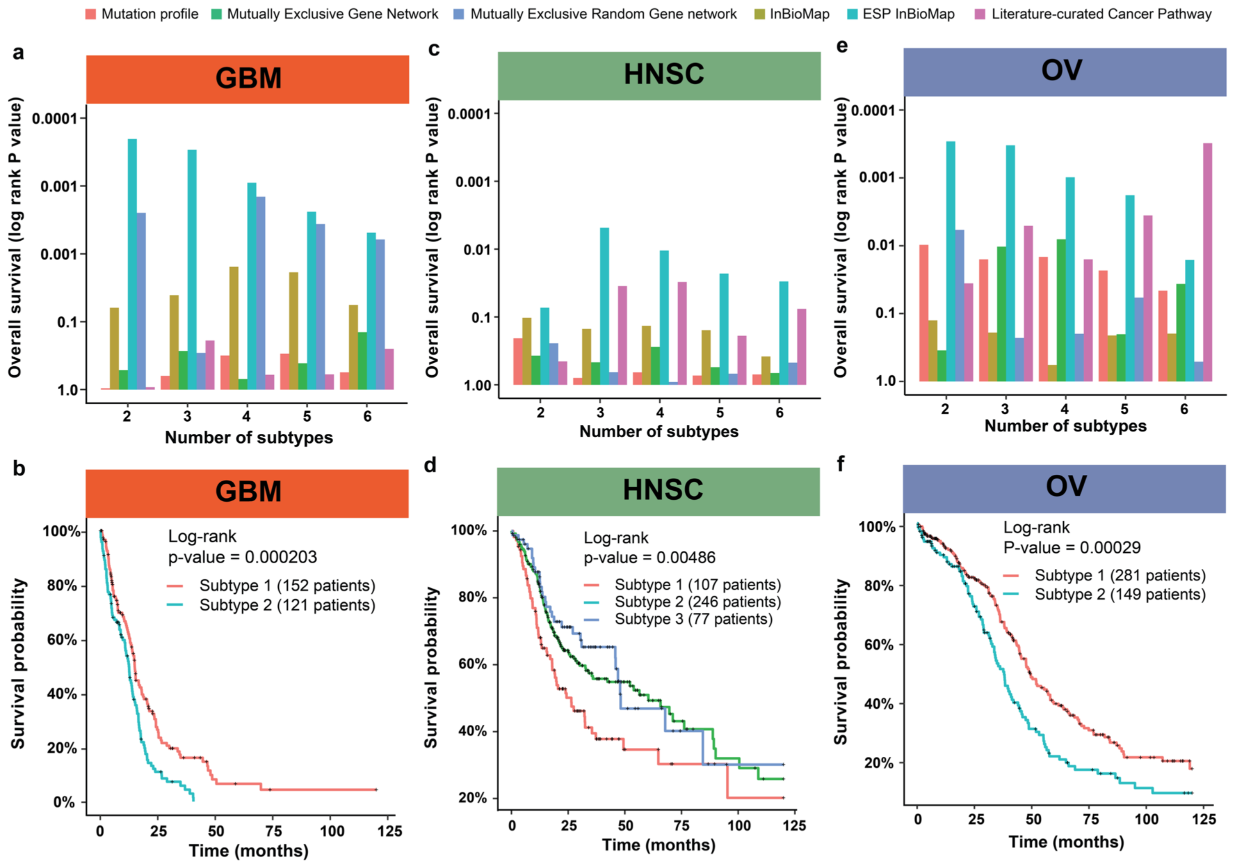

Supplementary Figure 4. Using ESPs to stratify Glioblastoma (GBM), Head and neck (HNSC HPV negative), Ovarian (OV) patients. a, Statistical significance of GBM patient stratification versus different numbers of subtypes. Statistical significance is determined by cox regression and log-rank test. **b,** Kaplan-Meier survival plot for GBM patients when stratified into two subtypes. **c,** Statistical significance of HNSC patient stratification versus different numbers of subtypes. Statistical significance is determined by cox regression and log-rank test. **d,** Kaplan-Meier survival plot for HNSC patients when stratified into three subtypes. **e,** Statistical significance of OV patient stratification versus different numbers of subtypes. Statistical significance is determined by cox regression and log-rank test. **f,** Kaplan-Meier survival plot for OV patients when stratified into two subtypes.

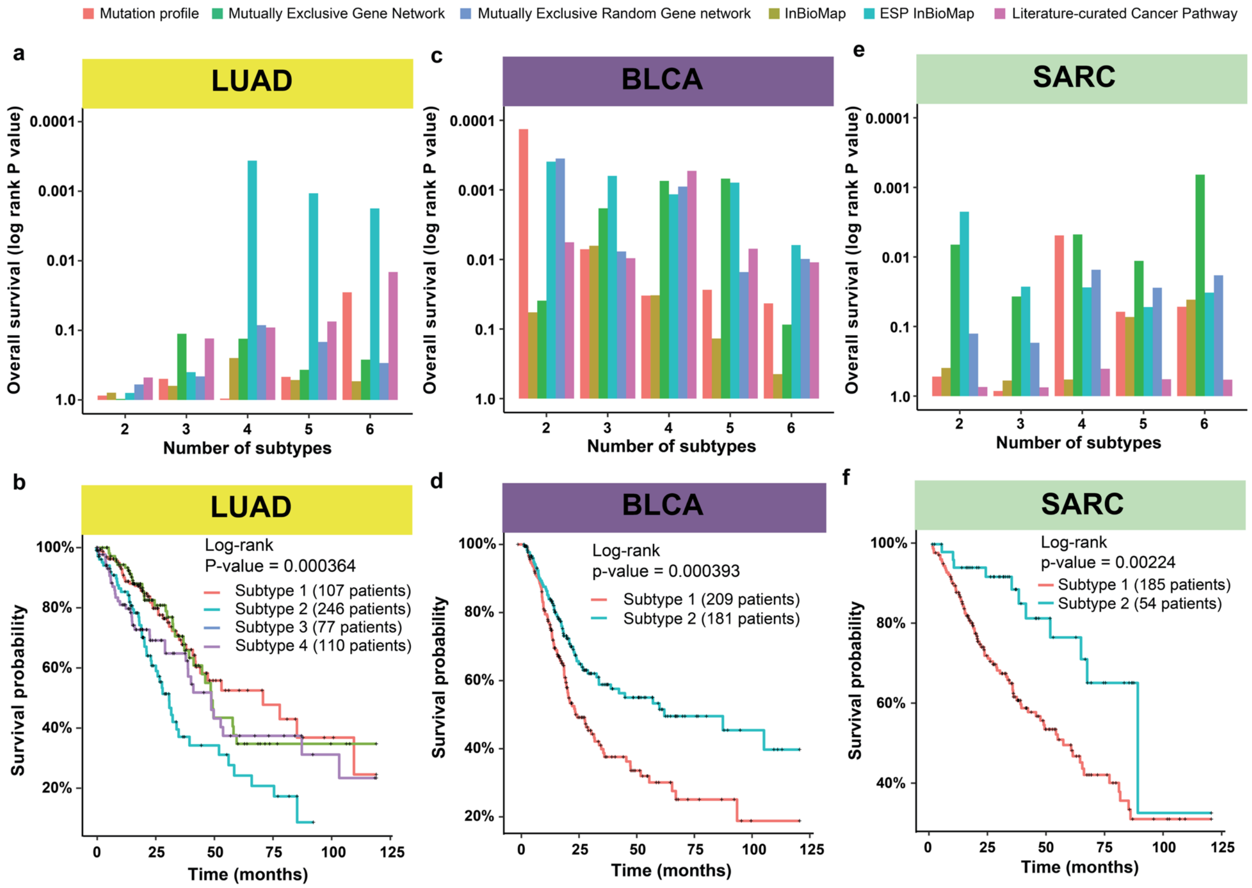

Supplementary Figure 5. Using ESPs to stratify Lung adenocarcinoma (LUAD), Bladder (BLCA), Sarcoma (SARC) cancer patients. a, Statistical significance of LUAD patient stratification versus different numbers of subtypes. Statistical significance is determined by cox regression and log-rank test. **b,** Kaplan-Meier survival plot for LUAD patients when stratified into four subtypes. **c,** Statistical significance of BLCA patient stratification versus different numbers of subtypes. Statistical significance is determined by cox regression and log-rank test. **d,** Kaplan-Meier survival plots for BLCA patients when stratified into two subtypes. **e,** Statistical significance of SARC patient stratification versus different numbers of subtypes. Statistical significance is determined by cox regression and log-rank test. **f,** Kaplan-Meier survival plot for SARC patients when stratified into two subtypes.

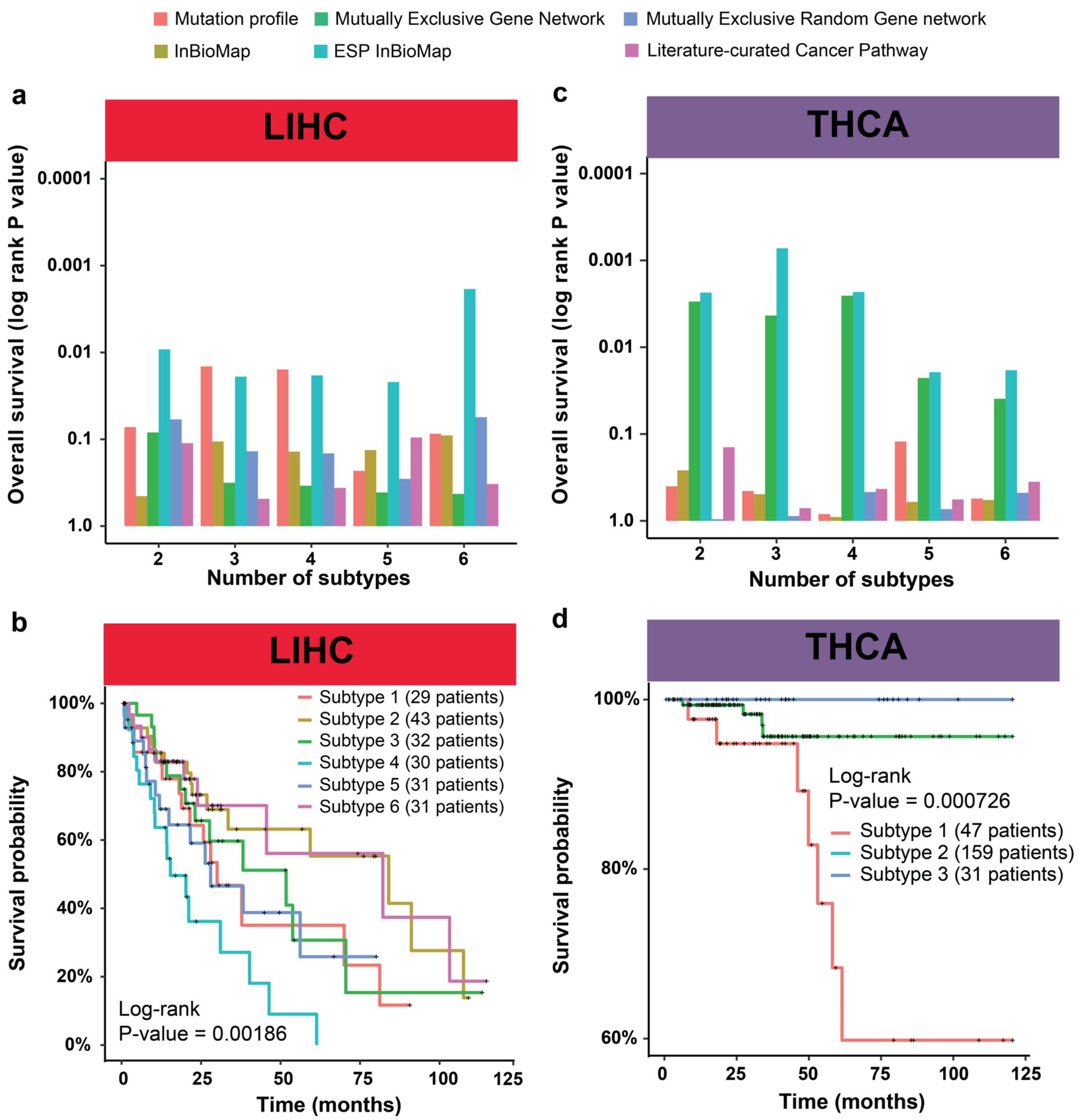

Supplementary Figure 6. Using ESPs to stratify Liver (LIHC), Thyroid (THCA) patients. a, Statistical significance of LIHC patient stratification versus different numbers of subtypes. Statistical significance is determined by cox regression and log-rank test. **b,** Kaplan-Meier survival plot for LIHC patients when stratified into six subtypes. **c,** Statistical significance of THCA patient stratification versus different numbers of subtypes. Statistical significance is determined by cox regression and log-rank test. **d,** Kaplan-Meier survival plot for THCA patients when stratified into three subtypes.

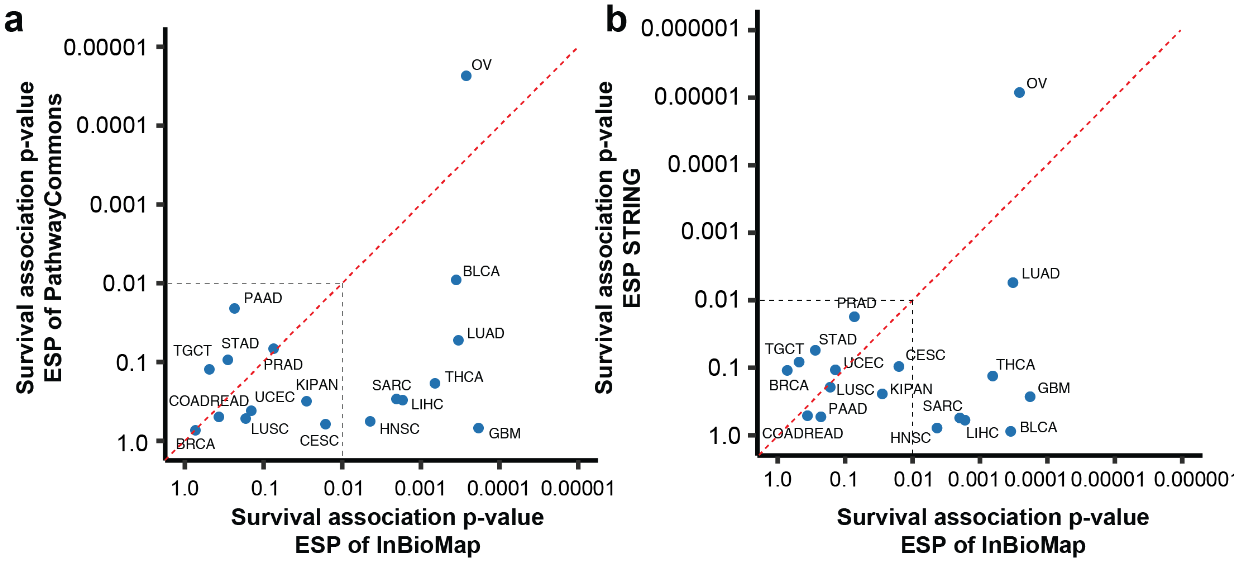

Supplementary Figure 7. Evaluation performance of different input sources. a, ESP derived from InBioMap versus ESP derived from PathwayCommons. **b**, ESP derived from InBioMap versus ESP derived from STRING.

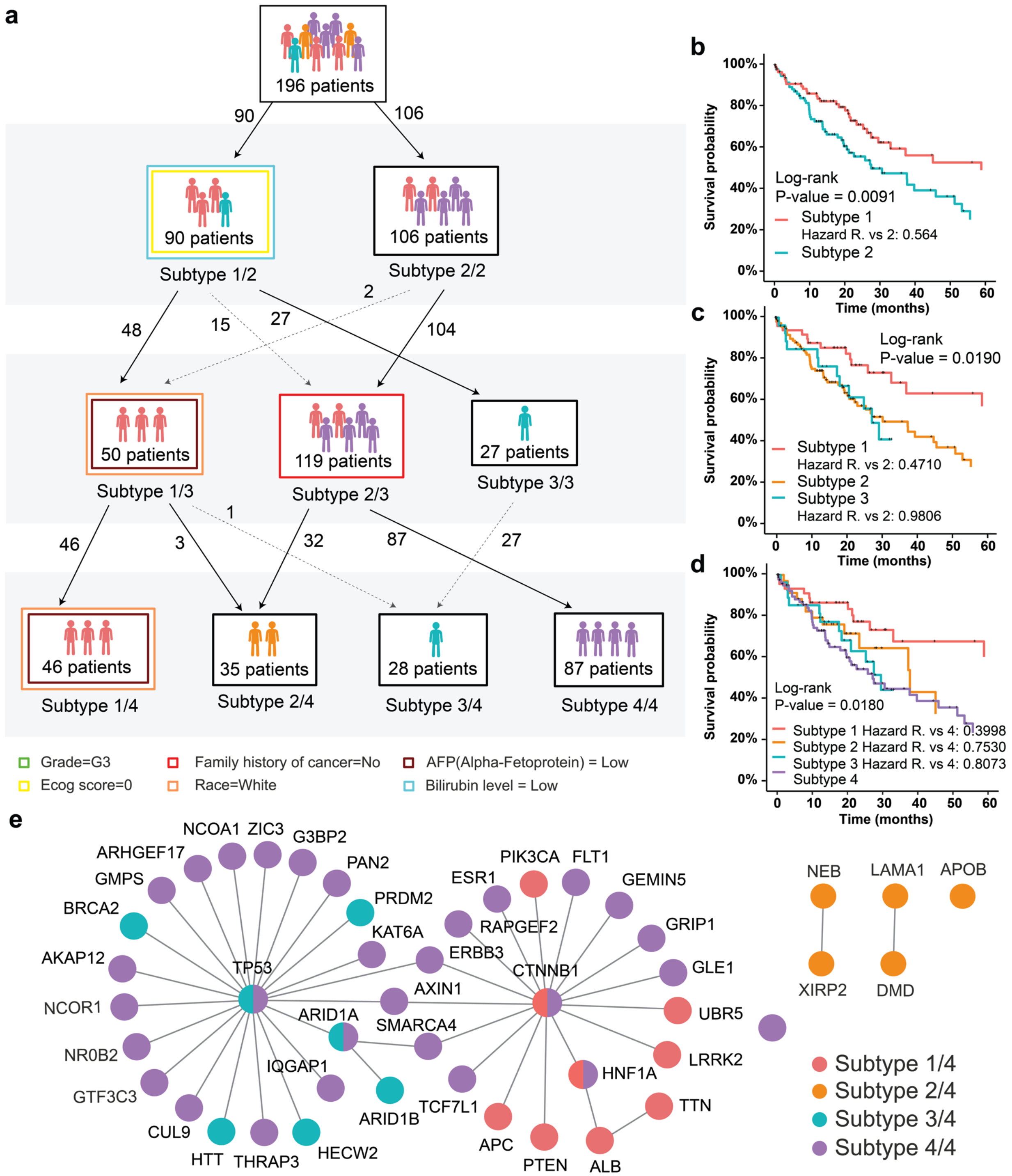

Supplementary Figure 8. Hierarchical clustering and characteristic ESP of liver cancer (LIHC) cancer patients in TCGA. **a**, Hierarchical organization of patients, at increasing resolution from 2-4 subtypes. **b-d**, Kaplan-Meier survival plots for different numbers of subtypes. **e**, Differentially mutated ESP used in assortment of patients into subtypes.

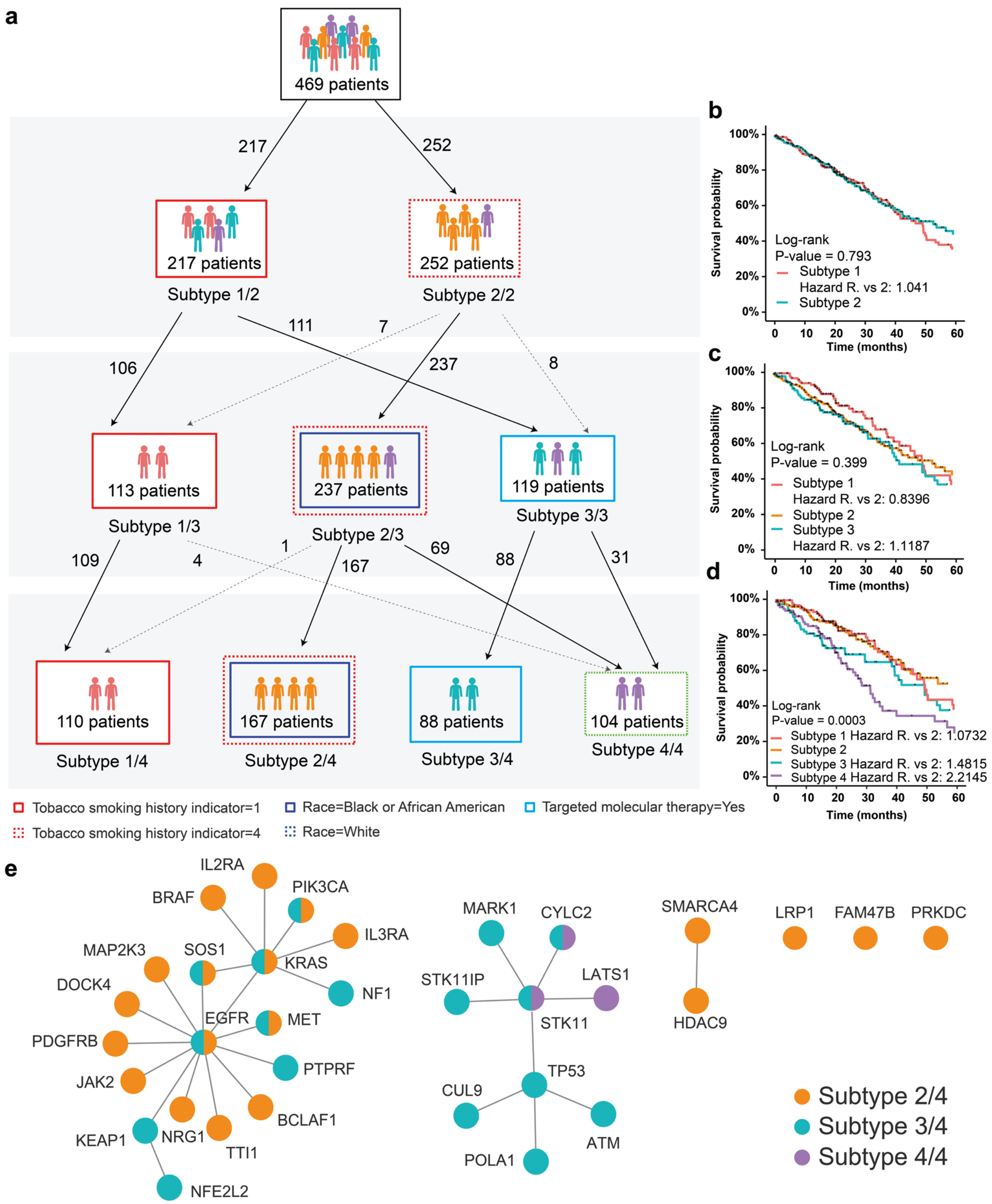

Supplementary Figure 9. Hierarchical clustering and characteristic ESP of lung adenocarcinoma (LUAD) patients in TCGA. **a**, Hierarchical organization of patients, at increasing resolution from 2-4 subtypes. **b-d**, Kaplan-Meier survival plots for different numbers of subtypes. **e**, Differentially mutated ESP used in assortment of patients into subtypes.

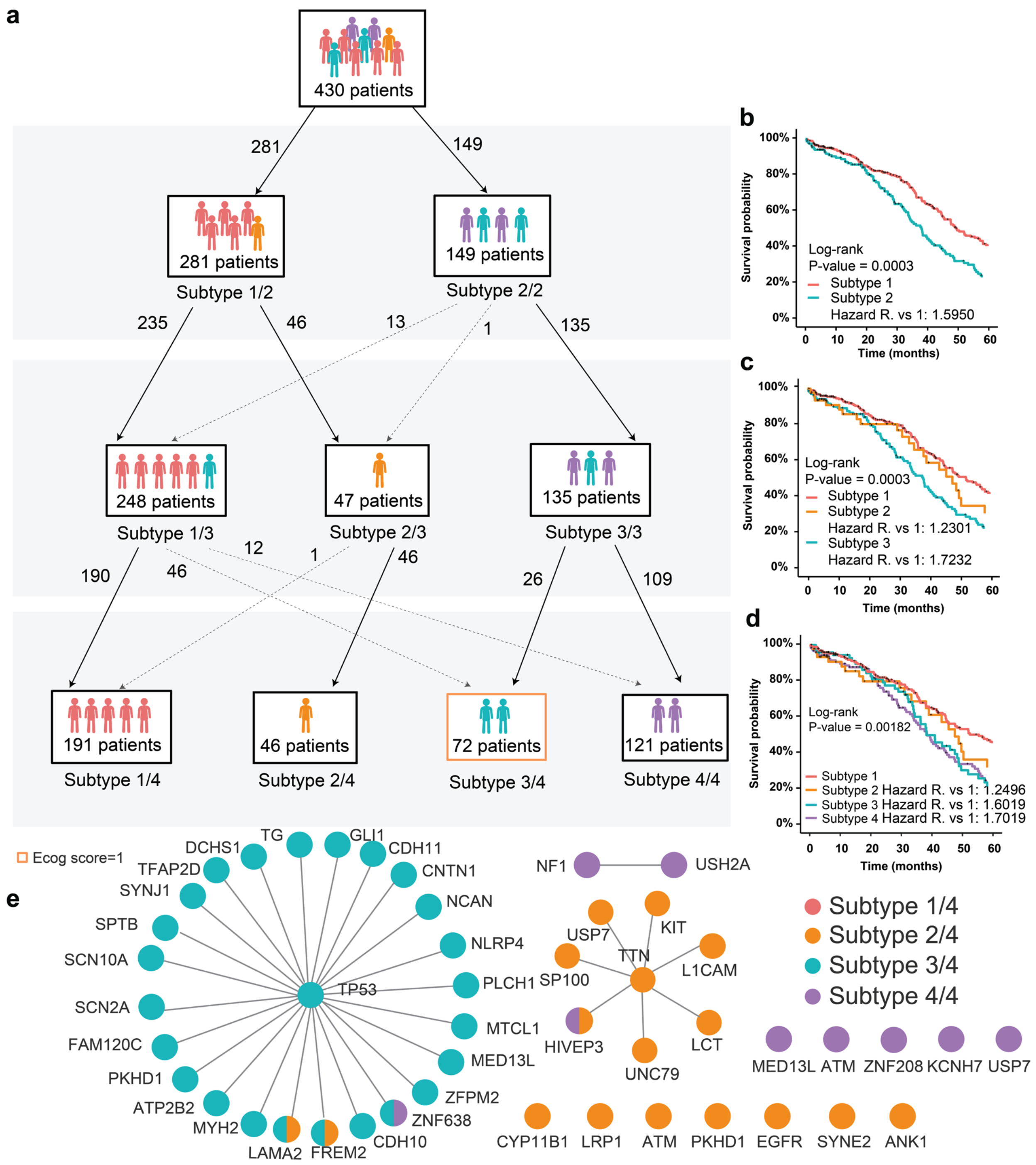

Supplementary Figure 10. Hierarchical clustering and characteristic ESP of ovarian cancer (OV) patients in TCGA. **a**, Hierarchical organization of patients, at increasing resolution from 2-4 subtypes. **b-d**, Kaplan-Meier survival plots for different numbers of subtypes. **e**, Differentially mutated ESP used in assortment of patients into subtypes.

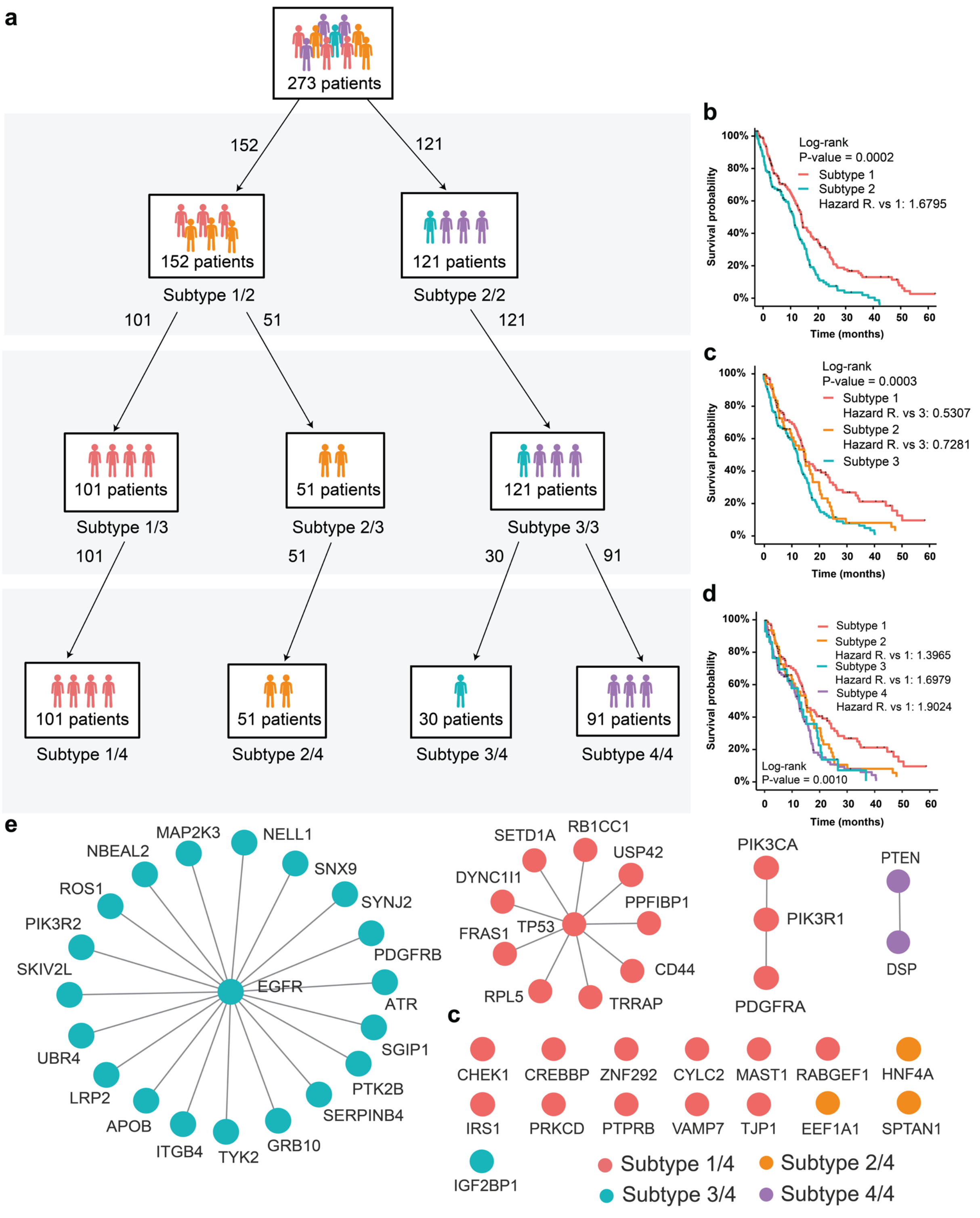

Supplementary Figure 11. Hierarchical clustering and characteristic ESP of glioblastoma (GBM) patients in TCGA. **a**, Hierarchical organization of patients, at increasing resolution from 2-4 subtypes. **b-d**, Kaplan-Meier survival plots for different numbers of subtypes. **e**, Differentially mutated ESP used in assortment of patients into subtypes.

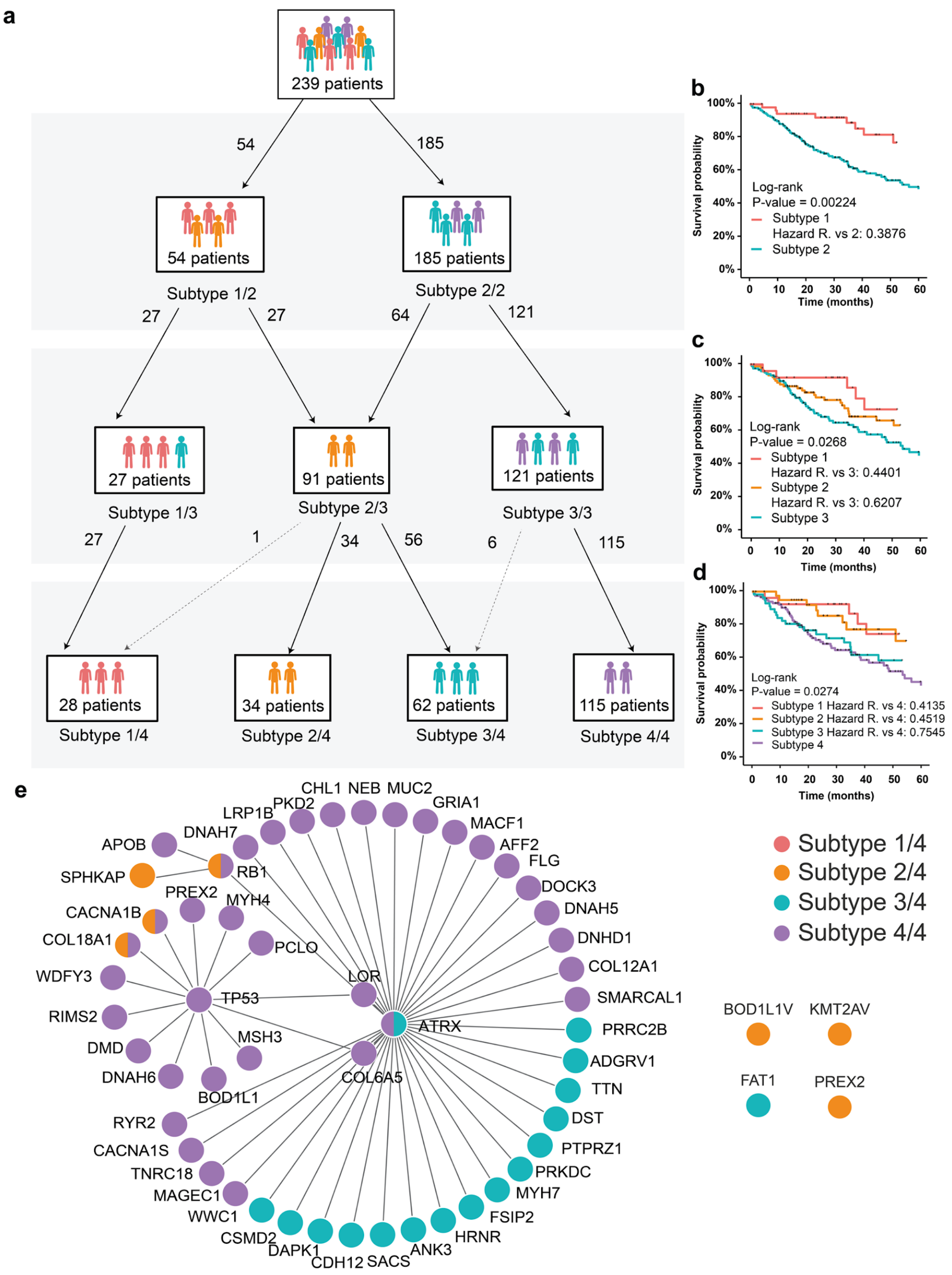

Supplementary Figure 12. Hierarchical clustering and characteristic ESP of sarcoma (SARC) patients in TCGA. **a**, Hierarchical organization of patients, at increasing resolution from 2-4 subtypes. **b-d**, Kaplan-Meier survival plots for different numbers of subtypes. **e**, Differentially mutated ESP used in assortment of patients into subtypes.

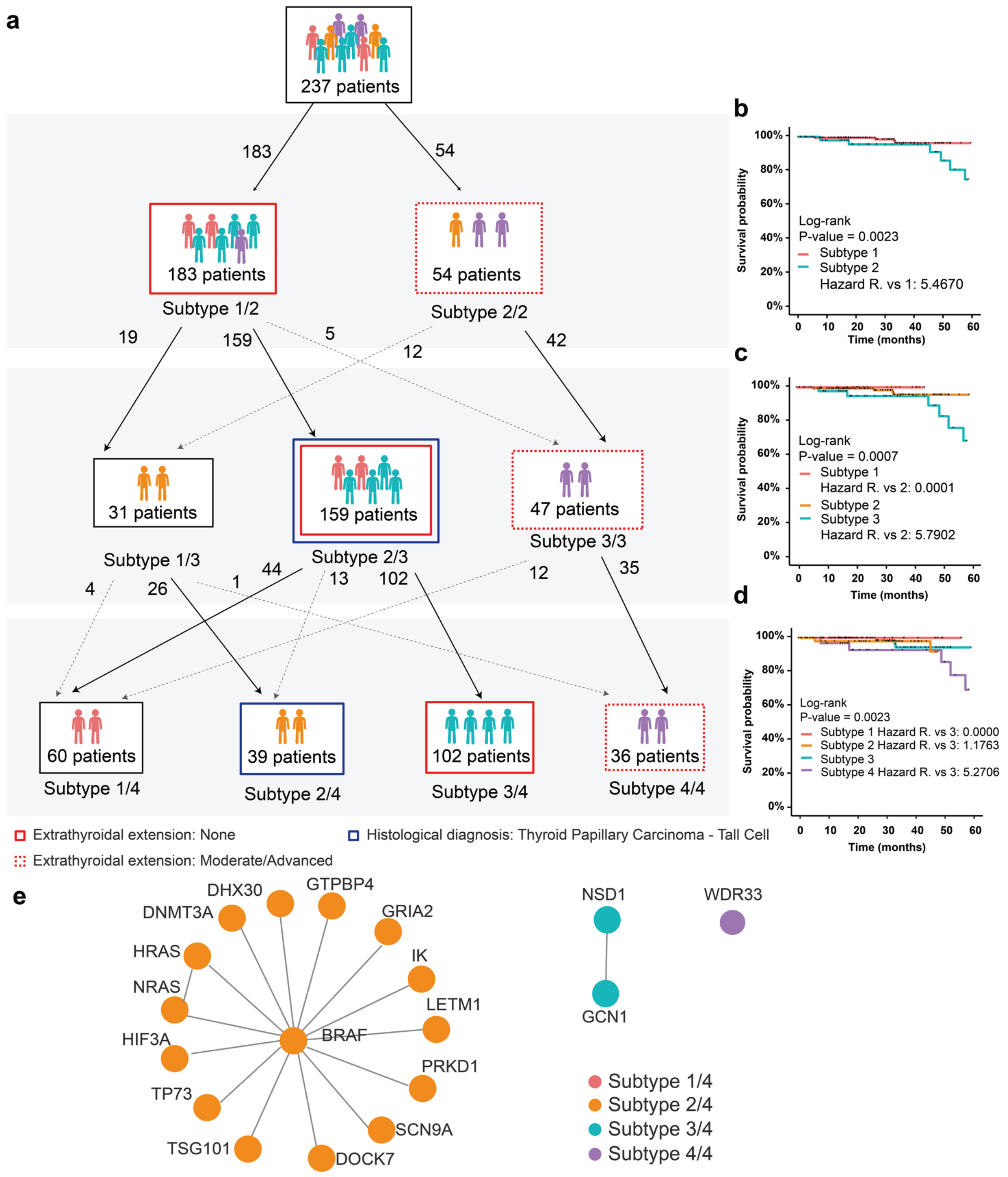

Supplementary Figure 13. Hierarchical clustering and characteristic ESP of thyroid (THCA) patients in TCGA. **a**, Hierarchical organization of patients, at increasing resolution from 2-4 subtypes. **b-d**, Kaplan-Meier survival plots for different numbers of subtypes. **e**, Differentially mutated ESP used in assortment of patients into subtypes.

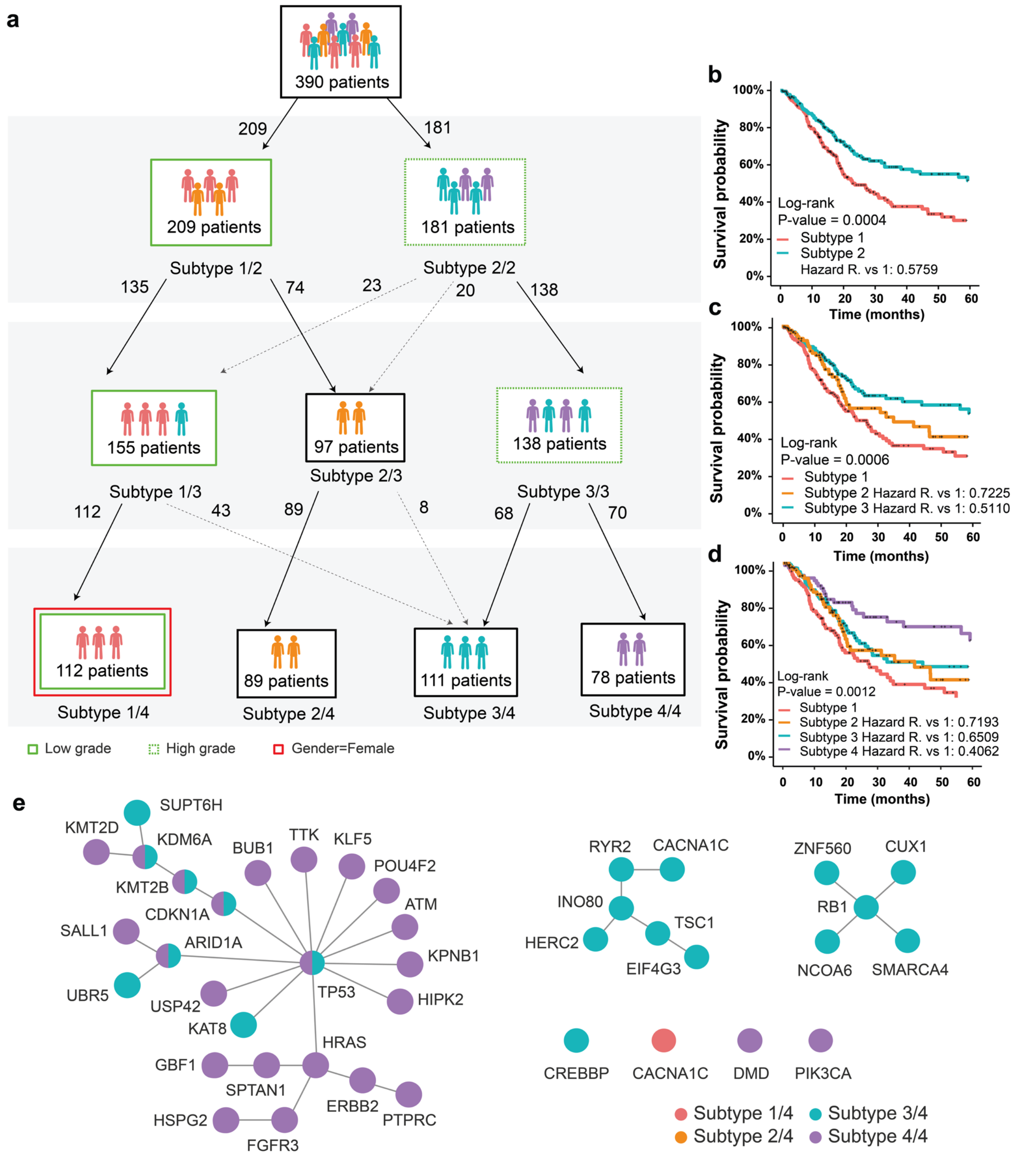

Supplementary Figure 14. Hierarchical clustering and characteristic ESP of bladder cancer (BLCA) patients in TCGA. **a**, Hierarchical organization of patients, at increasing resolution from 2-4 subtypes. **b-d**, Kaplan-Meier survival plots for different numbers of subtypes. **e**, Differentially mutated ESP used in assortment of patients into subtypes.

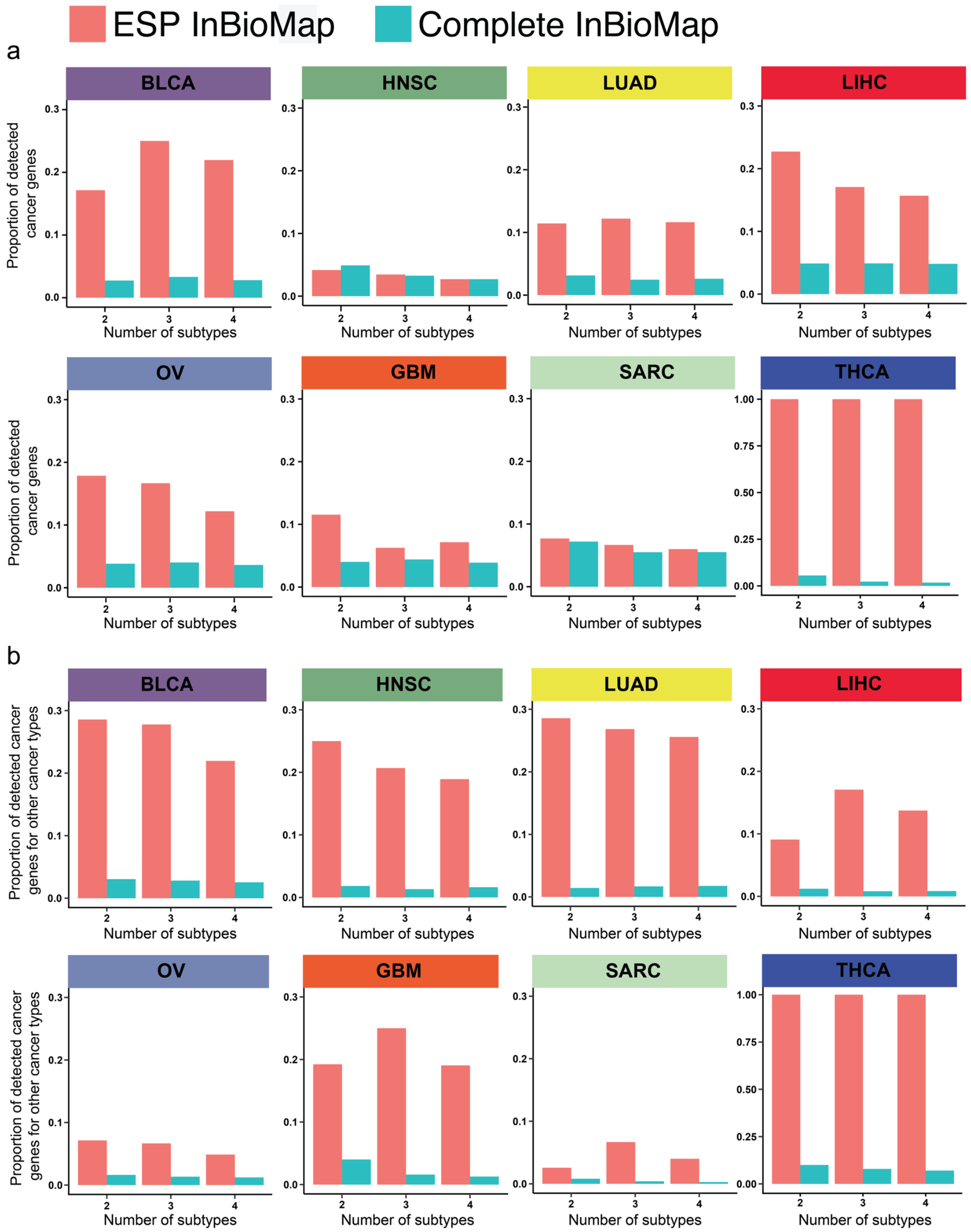

Supplementary Figure 15. Proportion of well-known cancer genes detected in ESP InBioMap verse complete InBioMap for 2-4 subtypes of 8 cancer types. a, Cancer genes were specific to cancer types. For example, for bladder cancer, only genes documented to be involved in bladder cancer were counted. **b,** Cancer genes were not specific to cancer types. For example, for bladder cancer, only genes documented to be involved in liver cancer were also counted.

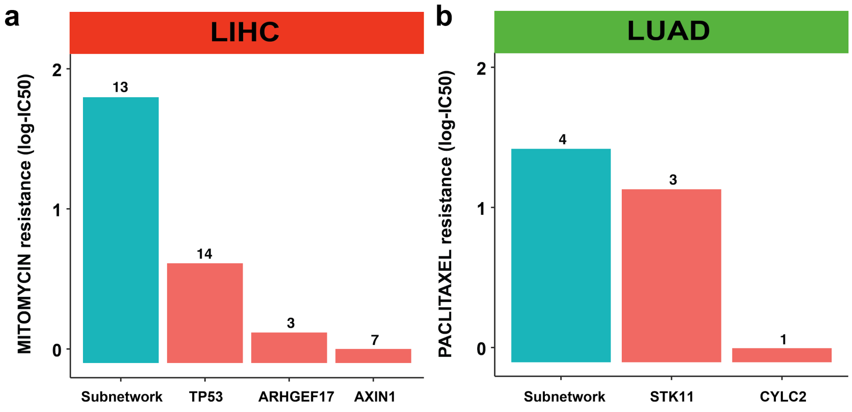

Supplementary Figure 16. Aggregation effects of ESPs in LIHC and LUAD. a, Drug resistance (log IC50) on cell line data using the aggregate of genes in the liver ESP versus each of its genes individually. **b**, Drug resistance (log IC50) on cell line data using the aggregate of genes in the lung ESP versus each of its genes individually.
