# Supplementary material for: Typing tumors using pathways selected by somatic evolution
Source: Nat Commun. 2018 Oct 8;9:4159. doi: 10.1038/s41467-018-06464-y (PMC6175900; doi:10.1038/s41467-018-06464-y)
Supplement: Supplementary file 3 — Description of Additional Supplementary Files [file 41467_2018_6464_MOESM3_ESM.pdf]

Description of Additional Supplementary Files

File Name: Supplementary Data 1
Description: **Evolutionarily selected pathway (ESP) map for 7 cancer types.**

File Name: Supplementary Data 2
Description: **Protein interactions involved in ESPs from multiple cancer types.**

File Name: Supplementary Data 3
Description: **Statistical association of ESP-based subtypes with known cancer subtypes and clinical measurements provided by TCGA.** P-value for known cancer subtypes was calculated by 2^2 test. P-value for clinical variables was calculated by one-sided Fisher exact test corrected by Bonferroni correction. P-value for additional predicting power for survival predictions is calculated by likelihood ratio test. See attached Excel spreadsheet.

File Name: Supplementary Data 4
Description: **Statistical association of ESP with cellular functions known to be associated with cancer.** P-value was calculated by one tail ranksum test.
